# Fertility-care readiness among healthcare students and workers in Al-Baha, Saudi Arabia: a cross-sectional study

**DOI:** 10.1186/s40834-026-00467-7

**Published:** 2026-07-22

**Authors:** Mohammad A. Albanghali

**Affiliations:** Department of Public Health, Faculty of Applied Medical Sciences, Al-Baha University, Al Baha, 65779 Saudi Arabia

**Keywords:** Fertility, Reproductive health, Family planning, Saudi Arabia

## Abstract

**Background:**

Saudi Arabia has experienced a marked fertility decline alongside health-system reforms under Vision 2030, yet evidence on healthcare workforce readiness for fertility and reproductive health (RH) counselling remains limited at the provincial level. Most Saudi studies have examined community demand, married women in primary care, or specialist groups in major urban centres. Demand-side evidence from Al-Baha indicates limited exposure to family-planning counselling and greater reliance on media than healthcare providers for RH information, highlighting the need to assess provider-side readiness in transitional provinces.

**Methods:**

A cross-sectional online survey was conducted from December 2024 to February 2025 among healthcare students enrolled at Al-Baha University and active healthcare workers employed by Al-Baha Health Cluster across Public Health, Medicine, Nursing, Pharmacy, and Laboratory Medicine. A 23-item instrument assessed four domains: Knowledge and Education, Access and Practice, Beliefs/Attitudes/Cultural Norms, and Lifestyle and Health Status.

**Results:**

Among 577 respondents, the mean age was 25.7 ± 9.0 years and 406 (70.4%) were students. Only 75 (13.0%) had attended a fertility/RH workshop, 126 (21.8%) reported full familiarity with international RH guidelines, and 209 (36.2%) were very confident in cross-cultural counselling. In multivariable analysis, high counselling confidence was associated with workshop attendance (adjusted OR [aOR] 10.78; 95% CI 4.92–23.62), cultural-value alignment with family planning (aOR 6.10; 95% CI 3.28–11.35), city residence (aOR 3.21; 95% CI 2.04–5.06), and above-median knowledge (aOR 1.71; 95% CI 1.05–2.79). Healthcare worker status showed a negative adjusted association (aOR 0.37; 95% CI 0.17–0.81), requiring cautious interpretation.

**Conclusion:**

Fertility-care readiness in Al-Baha was uneven and was associated mainly with modifiable factors, notably workshop attendance, cultural-value alignment with family planning, and knowledge level, as well as the contextual factor of urban residence, rather than professional role alone. Structured fertility/RH training, culturally responsive counselling education, and capacity building beyond urban centres may strengthen workforce readiness for RH counselling.

**Clinical trial number:**

Not applicable.

**Supplementary Information:**

The online version contains supplementary material available at https://doi.org/10.1186/s40834-026-00467-7.

## Background

Family planning and reproductive health are central to maternal and child wellbeing, gender equity, and sustainable development. Effective reproductive-health (RH) services support informed decision-making on fertility, contraception, and maternal care, and are associated with reductions in maternal and infant mortality [1, 2]. Globally, fertility rates have declined alongside expanded contraceptive access, improvements in women’s education, urbanisation, and economic development. However, inequities in access to services and counselling persist, particularly in rural and underserved settings [3–7]. 

Saudi Arabia has undergone one of the fastest demographic transitions in the region. The national total fertility rate has declined from more than six children per woman in the 1980s to approximately 2.3–2.5 by 2022, alongside substantial gains in female education and labour-force participation [8–10]. Vision 2030 and the Health Sector Transformation Program have further emphasised primary-care expansion and reproductive-health service development. These objectives are embedded in the Health Sector Transformation Program and are supported by national guidelines on family planning [11]. Despite this system-level progress, fertility-related counselling, cultural readiness for family-planning conversations, and equitable access in transitional regions remain insufficiently characterised. Inter-regional disparities in family-planning awareness, use, and unmet need have also been reported [12–14]. Strengthening workforce readiness at the provincial level is therefore important for supporting national RH objectives and improving access to informed, culturally appropriate counselling.

Fertility, contraception, and assisted-reproduction counselling require more than technical knowledge. They also depend on communication skills, awareness of referral pathways, and sensitivity to cultural and religious values. In Saudi Arabia, cultural and religious norms generally permit family planning while emphasising marital, familial, and ethical considerations. Accordingly, effective counselling requires providers to reconcile evidence-based professional guidance with local values and patient preferences [15–20]. However, most Saudi KAP research has focused on community samples, particularly married women in primary-care settings [13], or on contraceptive use among women in other Saudi provinces [14, 21], with limited attention to specialist groups in central urban regions [9]. Evidence on multidisciplinary healthcare students and professionals in semi-rural transitional provinces remains limited.

Recent demand-side evidence from Al-Baha highlights this gap. Albanghali et al. reported that only 23.5% of women in the province were aware of family-planning initiatives, 18.0% had ever received family-planning counselling, and media was a more common RH information source than healthcare workers (51.1% vs. 18.8%), despite 94.0% reporting access to a healthcare facility within thirty minutes [22]. These findings suggest that service availability alone may be insufficient if counselling is not routinely delivered, trusted, or recognised as a key source of RH information. The authors therefore called for stronger provider capacity and greater integration of family planning into routine primary care. This raises an important supply-side question: are the current and emerging healthcare workers expected to provide such counselling adequately prepared, and which factors are most closely associated with their readiness?

Workforce readiness for fertility and RH counselling is likely to reflect several interacting factors, including formal knowledge, exposure to training and guidelines, perceived service access, attitudinal alignment with family-planning principles, and the lifestyle behaviours that healthcare workers may model in practice [23]. However, provincial-level evidence on these provider-side factors, and on the modifiable determinants of counselling confidence, remains sparse. The present study therefore aimed to assess fertility-care readiness among healthcare students and active professionals in Al-Baha, Saudi Arabia, and to identify educational, cultural, and contextual factors associated with high self-rated confidence in fertility counselling. A secondary objective was to examine whether professional role remained associated with readiness and counselling confidence after accounting for demographic, educational, and contextual differences. While recent work has examined fertility awareness and parenthood intentions among physicians in Jazan [24], that study focused on physicians’ personal reproductive intentions rather than counselling readiness across disciplines. To our knowledge, the present study is the first to provide a comprehensive, multidisciplinary evaluation bridging both healthcare students and active professionals to identify the structural and modifiable determinants of fertility-counselling confidence in a transitional Saudi province. In addition, to explore the alignment between objective knowledge and self-rated confidence, respondents were classified into readiness-confidence profiles to identify patterns of miscalibration, specifically potential overconfidence among those with lower knowledge and under-confidence among those with higher knowledge, that may have implications for training design.

## Materials and methods

### Study design and setting

This was a cross-sectional online survey conducted in Al-Baha Province, Saudi Arabia, between 15 December 2024 and 28 February 2025. Al-Baha is a semi-rural southwestern province served by three regional general hospitals and a primary-healthcare network, with a demographic and infrastructural profile comparable to several other transitional Saudi provinces.

### Participants and sampling

Eligible participants were aged 18 years or older, resided in Al-Baha Province during the data-collection window, and belonged to one of two groups: [1] healthcare students enrolled in Public Health, Medicine, Nursing, Pharmacy, or Laboratory Medicine programmes at Al-Baha University; or healthcare workers employed by the Al-Baha Health Cluster (Ministry of Health) across the same five disciplines. Convenience sampling was used; the questionnaire was distributed through institutional email lists and WhatsApp groups maintained by the university and the health cluster, with voluntary, anonymous participation and electronic informed consent. No comprehensive invitation registers or total eligible headcount was available to the investigators prior to data collection. To minimise duplicate entries, the online platform was configured to restrict repeat submissions without retaining identifiable usernames in the analytic dataset. In addition, prior to analysis, all responses were screened for exact-duplicate records across all variables; responses identical across all fields were treated as accidental resubmissions and retained only once.

### Survey instrument and domains

A 23-item questionnaire was developed based on validated tools used in prior Saudi and regional KAP literature [12–14, 21, 25]. Prior to deployment, the instrument was reviewed for face and content validity by a panel of three academic public-health specialists. A pilot administration was conducted among 10 participants not included in the final sample to assess clarity, comprehension, and item acceptability; minor wording adjustments were made based on feedback. The questionnaire was administered in English, which is the language of instruction in the affiliated health sciences programmes. No translation was required. The scored items were grouped into four domains: Knowledge and Education (K; 7 items), Access and Practice (AP; 4 items), Beliefs/Attitudes/Cultural Norms (BAC; 7 items), and Lifestyle and Health Status (LHS; 5 items). Each item was coded so that higher values reflected greater fertility-care readiness within the relevant domain. Domain scores were calculated by summing coded item responses within Knowledge and Education, Access and Practice, Beliefs/Attitudes/Cultural Norms, and Lifestyle and Health Status. The pooled readiness score was calculated by summing all 23 coded items. Higher scores indicated greater readiness. Item coding and score ranges are provided in the supplementary scoring table. Cronbach’s α was 0.81 for the Knowledge subscale, 0.06 for Access and Practice (which combines four conceptually distinct facets of access and practice), 0.48 for Beliefs/Attitudes/Cultural Norms, 0.46 for Lifestyle and Health Status, and 0.80 for the pooled 23-item scale. Because internal consistency was modest for the AP, BAC, and LHS subscales, item-level findings are reported alongside domain scores throughout to preserve transparency, rather than relying solely on composite subscale totals.

### Outcomes and explanatory variables

The primary outcome was high self-rated counselling confidence, defined as reporting being “very confident” in cross-cultural fertility counselling. This variable was modelled as a binary outcome, coded 1 for “very confident” and 0 for all other responses. Self-rated confidence was not interpreted as objective counselling competence. Secondary outcomes included the four domain scores and strong endorsement of curriculum integration. Explanatory variables were grouped into three categories: demographic and contextual variables, including age, marital status, education, residence, body mass index (BMI), speciality, and professional role; educational and readiness variables, including workshop attendance, guideline familiarity, perceived curriculum coverage, and Knowledge score; and cultural-attitudinal variables, including cultural-value alignment with family planning, professional acceptability, and perceived cultural influence on family planning.

Readiness-confidence profiles were created by cross-classifying respondents according to knowledge-domain score and self-rated counselling confidence. Respondents with above-median knowledge and very high confidence were classified as calibrated high-readiness; those with above-median knowledge but lower confidence as under-confident; those with below-median knowledge but very high confidence as potentially overconfident; and those with below-median knowledge and lower confidence as low-readiness.

### Statistical analysis

Analyses used IBM SPSS Statistics 21.0. Continuous variables are summarised as mean ± SD and median (IQR); categorical variables as n (%). Group differences were tested using Mann-Whitney U, Kruskal-Wallis, and Pearson’s χ² with Cramér’s V. The analysis plan was hierarchical: descriptive statistics for the whole sample; crude comparisons by professional role; and univariable and multivariable logistic regression for high counselling confidence. To assess multicollinearity in the multivariable model, Variance Inflation Factors (VIF) were computed for all predictors; a VIF below 5 was considered acceptable. To examine the trajectory of the healthcare worker coefficient across model specifications, a hierarchical two-block logistic regression was performed: Block 1 included demographic and structural variables (role, age, residence, speciality), and Block 2 added educational and cultural mediators (workshop attendance, cultural-value alignment, knowledge score, guideline familiarity). The change in model fit between blocks was assessed with a likelihood-ratio test. Crude role comparisons were treated as descriptive only because students and workers differed substantially in age, education, marital status, residence, and BMI. As sensitivity checks, the role contrast was repeated within the university-educated subset (*n* = 451) and through linear regression of each domain on role with adjustment for the same demographic covariates. A two-sided *p* < 0.05 was the significance threshold. No a priori sample size was calculated, as the eligible sampling frame, healthcare students at Al-Baha University and active professionals at the Al-Baha Health Cluster across the five target disciplines, could not be precisely enumerated from available institutional records prior to data collection. Post hoc, with *n* = 577 and assuming a proportion of 0.50 (most conservative) at the 95% confidence level, the achieved sample provides a margin of error of ± 4.1% points using the standard formula for large or unknown populations [26]. This level of precision is considered adequate for a provincial workforce survey of this scope.

### Ethics

The study was approved by the Deanship of Scientific Research, Al-Baha University (Approval No. 46110701 − 20241117) and was conducted in accordance with the Declaration of Helsinki. No personally identifiable information was collected.

## Results

### Participants’ characteristics

A total of 577 healthcare students and active professionals took part. The mean age was 25.7 ± 9.0 years (median 22; IQR 21 to 25) and the mean body-mass index was 23.3 ± 5.4 kg/m². Students accounted for 70.4% (*n* = 406) of the sample and active healthcare workers 29.6% (*n* = 171). The two subgroups differed across all measured baseline characteristics examined (Table 1): workers were older (median 34 vs. 21 years), more often married (63.2% vs. 5.2%), more often residents of cities (75.4% vs. 43.1%), and more often overweight or obese (50.9% vs. 20.0%). The five disciplines were represented in roughly balanced proportions: Public Health 27.9%, Medicine 20.6%, Nursing 20.3%, Pharmacy 17.7%, and Laboratory Medicine 13.5%. These baseline imbalances should be considered when interpreting subsequent role comparisons. (Table 1)

**Table 1:** Sociodemographic characteristics overall and by professional role (Student vs. Healthcare Worker) among healthcare students and professionals in Al-Baha Province, Saudi Arabia (*N* = 577)

Variable	Overall (*N* = 577)	Students (*n* = 406)	Healthcare workers (*n* = 171)	*p*	V
**Age (years)**,** mean ± SD**	25.7 ± 9.0	21.6 ± 3.2	35.5 ± 10.7	< 0.001	
**BMI (kg/m²)**,** mean ± SD**	23.3 ± 5.4	21.9 ± 4.7	26.6 ± 5.6	< 0.001	
***Marital status***					
Single	430 (74.5)	385 (94.8)	45 (26.3)	< 0.001	0.716
Married	129 (22.4)	21 (5.2)	108 (63.2)		
Widowed	12 (2.1)	0 (0.0)	12 (7.0)		
Divorced	3 (0.5)	0 (0.0)	3 (1.8)		
***Education level***					
Secondary	60 (10.4)	57 (14.0)	3 (1.8)	< 0.001	0.498
University	451 (78.2)	343 (84.5)	108 (63.2)		
Postgraduate	66 (11.4)	6 (1.5)	60 (35.1)		
***Speciality***					
Public Health	161 (27.9)	128 (31.5)	33 (19.3)	< 0.001	0.333
Medicine	119 (20.6)	98 (24.1)	21 (12.3)		
Nursing	117 (20.3)	60 (14.8)	57 (33.3)		
Pharmacy	102 (17.7)	84 (20.7)	18 (10.5)		
Laboratory Medicine	78 (13.5)	36 (8.9)	42 (24.6)		
***Years of experience in healthcare***					
<1 year	33 (5.7)	0 (0.0)	33 (19.3)		
1 to 5 years	31 (5.4)	0 (0.0)	31 (18.1)		
6 to 10 years	107 (18.5)	0 (0.0)	107 (62.6)		
Not applicable (students)	406 (70.4)	406 (100.0)	0 (0.0)		
***Residence (Governorate)***					
Al-Baha	242 (41.9)	167 (41.1)	75 (43.9)	0.002	0.173
Baljurashi	113 (19.6)	74 (18.2)	39 (22.8)		
Al-Aqiq	78 (13.5)	63 (15.5)	15 (8.8)		
Al-Makhwah	24 (4.2)	24 (5.9)	0 (0.0)		
Other	120 (20.8)	78 (19.2)	42 (24.6)		
***Type of residence***					
City	304 (52.7)	175 (43.1)	129 (75.4)	< 0.001	0.292
Village	273 (47.3)	231 (56.9)	42 (24.6)		
***BMI category***					
Underweight (< 18.5)	98 (17.0)	92 (22.7)	6 (3.5)	< 0.001	0.385
Healthy weight (18.5 to 24.9)	311 (53.9)	233 (57.4)	78 (45.6)		
Overweight (25.0 to 29.9)	93 (16.1)	57 (14.0)	36 (21.1)		
Obesity (≥ 30)	75 (13.0)	24 (5.9)	51 (29.8)		

Data are n (%) for categorical variables and mean ± SD for continuous variables. P-values are from Pearson’s χ² (categorical) and Mann-Whitney U (continuous), two-sided. Cramér’s V (V) is an effect-size measure for χ² associations. Students and healthcare workers differed on every demographic indicator measured, including age, marital status, education, residence, and BMI. Unadjusted contrasts between role groups in this and subsequent tables therefore should not be interpreted as independent role effects; adjusted analyses for the primary outcome are reported in Section “Factors associated with high counselling confidence” and Table 4. BMI = body mass index

### Item-level readiness across the four domains

Item-level distributions are presented in Table 2 and reveal several readiness gaps relevant to fertility counselling. Workshop exposure was limited: only 13.0% of respondents had ever attended a fertility/RH workshop. Familiarity with international RH guidelines was full for 21.8% and partial for the remainder, and full awareness of fertility-related services in the local area was reported by only 13.5%. Within Knowledge, 22.4% considered fertility comprehensively included in their training while 30.2% considered the coverage insufficient; the Knowledge domain scored 15.42 ± 3.65 (median 15; IQR 13 to 18; α = 0.81). Within Access and Practice, 76.6% believed family-planning tools were locally available and 85.6% reported no challenges in obtaining contraceptives; Among 129 married participants, 53.5% reported using a contraceptive method, most often oral contraceptive pills or intrauterine devices, while 46.5% reported no current method or did not report a method. Only 20.6% considered IVF availability sufficient. Within Beliefs/Attitudes/Cultural Norms, 81.8% rated family planning as fully or partially professionally acceptable and 75.6% as fully or partially aligned with personal cultural values, while 92.7% strongly endorsed the role of healthcare workers in promoting awareness; 69.5% identified three to four children as the local cultural ideal, and 90.6% rated delayed childbearing as having a major or moderate effect. Within Lifestyle, 50.8% partially followed an RH-supportive lifestyle, 95.8% did not use tobacco, and 18.2% reported a relevant condition such as diabetes or PCOS. Domain scores were AP 4.02 ± 0.93, BAC 18.16 ± 2.47, LHS 13.17 ± 1.92; pooled overall 50.56 ± 6.90 (α = 0.80). Just over a third of respondents (36.2%) rated themselves as very confident in cross-cultural fertility counselling.

**Table 2:** Item-level responses across knowledge and education, access and practice, beliefs/attitudes/cultural norms, and lifestyle and health status domains, among healthcare students and professionals in Al-Baha Province, Saudi Arabia (*N* = 577 unless otherwise noted)

Domain	Item & response	*n* (%)
Knowledge & Education (K)	**K1: Self-rated knowledge of fertility and RH**	
	• Moderate knowledge	229 (39.7)
	• High knowledge	171 (29.6)
	• Low knowledge	105 (18.2)
	• I don’t know	72 (12.5)
	**K2: Fertility awareness adequately included in education**	
	• Yes, but briefly	209 (36.2)
	• No, not sufficient	174 (30.2)
	• Yes, comprehensively	129 (22.4)
	• Not included	65 (11.3)
	**K3: Importance of RH education for healthcare students and professionals**	
	• Very important	440 (76.3)
	• Important	98 (17.0)
	• Moderately important	24 (4.2)
	• Not important	15 (2.6)
	**K4: Familiar with international RH guidelines (WHO**,** ASRM)**	
	• To some extent	267 (46.3)
	• No	184 (31.9)
	• Yes	126 (21.8)
	**K5: Attended fertility/RH workshops or training**	
	• No	299 (51.8)
	• Not yet, but interested	203 (35.2)
	• Yes	75 (13.0)
	**K6: Awareness of assisted reproductive technologies (IVF)**	
	• Moderate awareness	284 (49.2)
	• Not aware	179 (31.0)
	• High awareness	114 (19.8)
	**K7: Aware of fertility-related healthcare services in your area**	
	• Not aware	250 (43.3)
	• Partially aware	249 (43.2)
	• Yes, fully aware	78 (13.5)
Access & Practice (AP)	**AP1: Believe family-planning tools are available in your area**	
	• Yes	442 (76.6)
	• No	135 (23.4)
	**AP2: Encountered challenges in obtaining contraceptive methods**	
	• No	494 (85.6)
	• Yes, lack of knowledge about options	38 (6.6)
	• Yes, limited availability	18 (3.1)
	• Yes, high cost	15 (2.6)
	• Yes, social restrictions	12 (2.1)
	**AP3: Currently using any contraceptive method (married**, ***n*** **= 129)**	
	• Oral contraceptive pills	27 (20.9)
	• Intrauterine device (IUD)	24 (18.6)
	• Natural methods	12 (9.3)
	• Condoms	6 (4.7)
	• None / not reported	60 (46.5)
	**AP4: Availability of fertility treatments (IVF) sufficient in Saudi Arabia**	
	• Not sure	335 (58.1)
	• No	123 (21.3)
	• Yes	119 (20.6)
Beliefs, Attitudes & Cultural Norms (BAC)	**BAC1: Family planning acceptable from professional perspective**	
	• Fully acceptable	277 (48.0)
	• Partially acceptable	195 (33.8)
	• Neutral	90 (15.6)
	• Not acceptable	15 (2.6)
	**BAC2: Influence of culture/society on family planning in Al-Baha**	
	• Neutral	224 (38.8)
	• Moderately positive effect	206 (35.7)
	• Very positive effect	114 (19.8)
	• Very negative effect	33 (5.7)
	**BAC3: Healthcare workers have a role in promoting awareness**	
	• Yes, a major role	409 (70.9)
	• Yes, a minor role	126 (21.8)
	• No role	42 (7.3)
	**BAC4: Family-planning values compatible with own cultural values**	
	• Fully aligned	248 (43.0)
	• Partially aligned	188 (32.6)
	• Neutral	120 (20.8)
	• Not aligned	21 (3.6)
	**BAC5: Believe fertility rates in Al-Baha are declining**	
	• Not sure	391 (67.8)
	• Yes	105 (18.2)
	• No	81 (14.0)
	**BAC6: Ideal number of children in the local culture**	
	• 3 to 4	401 (69.5)
	• 1 to 2	92 (15.9)
	• 5 or more	84 (14.6)
	**BAC7: Effect of delayed childbearing on reproductive health**	
	• Major effect	275 (47.7)
	• Moderate effect	248 (43.0)
	• No effect	54 (9.4)
Lifestyle & Health Status (LHS)	**LHS1: Follow lifestyle that supports reproductive health**	
	• Partially	293 (50.8)
	• No	170 (29.5)
	• Yes, completely	114 (19.8)
	**LHS2: Frequency of physical activity**	
	• Rarely	236 (40.9)
	• Several times per week	224 (38.8)
	• Daily	81 (14.0)
	• I never exercise	36 (6.2)
	**LHS3: Smoke or use tobacco products**	
	• No	553 (95.8)
	• Yes	24 (4.2)
	**LHS4: Diet quality in terms of supporting reproductive health**	
	• Good	231 (40.0)
	• Moderate	223 (38.6)
	• Poor	63 (10.9)
	• Excellent	60 (10.4)
	**LHS5: Experienced health conditions (diabetes**,** PCOS)**	
	• No	472 (81.8)
	• Yes	105 (18.2)

Data are presented as n (%); column percentages are based on *N* = 577 except for AP3 (current contraceptive use), restricted to married respondents (*n* = 129). Domain abbreviations: K, Knowledge & Education; AP, Access & Practice; BAC, Beliefs, Attitudes & Cultural Norms; LHS, Lifestyle & Health Status. ART = assisted reproductive technologies; IVF = in vitro fertilisation; PCOS = polycystic ovary syndrome

### Crude variation in domain scores by role, training, and cultural strata

Crude domain scores varied widely across examined stratifiers (Table 3). Healthcare workers showed higher crude median scores than students on every domain (Knowledge 18 vs. 14; BAC 19 vs. 18; Lifestyle 14 vs. 13; pooled overall 53 vs. 50; all *p* < 0.01). However, students and workers differed markedly on every demographic in Table 1: only 6 of 406 students were aged over 25 (vs. 123 of 171 workers), and only 6 students held a postgraduate qualification (vs. 60 workers). Restricting the comparison to university-educated respondents (*n* = 451) attenuated the Lifestyle and Access differences (*p* = 0.052 and 0.92). After adjustment for age, education, marital status, residence, and BMI, professional role was no longer independently associated with domain scores (Knowledge β = -0.24, 95% CI -1.12 to + 0.65, *p* = 0.60; AP β = -0.06, *p* = 0.63; BAC β = -0.52, *p* = 0.12; Lifestyle β = -0.43, *p* = 0.11). The crude role pattern therefore appeared to reflect demographic and exposure differences rather than role status itself. Domain-score comparisons across all strata in this section are presented as descriptive findings only; given the modest internal consistency of the AP, BAC, and LHS subscales, they serve to contextualise item-level patterns and inform the selection of variables for the primary regression analysis, not to support independent inferential conclusions about domain-level readiness.

Across the remaining strata, Public Health respondents had the lowest median Knowledge score (13; IQR 12 to 16) compared with 15 to 16 for the other disciplines (*p* < 0.001), and city respondents scored higher than village respondents on Knowledge (median 16 vs. 14; *p* < 0.001). Workshop attendance and cultural-value alignment showed the largest domain-specific differences. Respondents who had attended a fertility/RH workshop had a higher Knowledge median score than those who had not attended, 21 (IQR 19–23) vs. 15 (IQR 12–17), *p* < 0.001. Similarly, respondents whose cultural values were fully or partially aligned with family planning had a higher BAC median score than those who were neutral or not aligned, 19 (IQR 18–20) vs. 16 (IQR 14–17), *p* < 0.001.

**Table 3:** Median (interquartile range) domain scores and pooled overall scores across role, training, and cultural strata, with non-parametric tests of association (*N* = 577)

Stratifiers	K	AP	BAC	LHS	Overall
**Role**					
Student (*n* = 406)	14 (12–17)	4 (3–4)	18 (16–20)	13 (12–14)	50 (45–54)
Healthcare workers (*n* = 171)	18 (16–21)	4 (3–5)	19 (18–20)	14 (13–15)	53 (49–58)
*p (MWU*,* crude)*	*< 0.001*	*0.003*	*< 0.001*	*< 0.001*	*< 0.001*
*p (adjusted*,* see text)*	*0.60*	*0.63*	*0.12*	*0.11*	*see text*
***Age group***					
≤25 years (*n* = 444)	14 (12–17)	4 (3–4)	18 (16–20)	13 (12–14)	49 (45–53)
>25 years (*n* = 129)	18 (16–22)	4 (3–5)	19 (18–21)	14 (13–15)	55 (50–59)
*p (MWU)*	*< 0.001*	*< 0.001*	*< 0.001*	*< 0.001*	*< 0.001*
***Speciality***					
Public Health (*n* = 161)	13 (12–16)	4 (3–5)	18 (17–20)	13 (12–14)	49 (45–53)
Medicine (*n* = 119)	16 (13–18)	4 (4–5)	18 (16–20)	13 (12–14)	53 (48–57)
Nursing (*n* = 117)	16 (15–18)	4 (3–5)	19 (17–20)	13 (12–14)	50.5 (46–56)
Pharmacy (*n* = 102)	15.5 (12–17)	4 (3–4)	18 (17–20)	13 (12–15)	50 (46–53)
Laboratory Medicine (*n* = 78)	15 (13–18)	4 (3–5)	19 (17–21)	13.5 (12–14)	54 (49–56)
*p (KW)*	*< 0.001*	*< 0.001*	*0.033*	*0.214*	*< 0.001*
***Type of residence***					
City (*n* = 304)	16 (13–18)	4 (3–5)	19 (17–20)	13 (12–14)	51 (46–57)
Village (*n* = 273)	14 (12–17)	4 (3–4)	18 (16–20)	13 (12–14)	50 (46–54)
*p (MWU)*	*< 0.001*	*0.022*	*0.058*	*0.033*	*0.283*
***BMI category***					
Underweight (*n* = 98)	14 (12–16)	4 (4–4)	19 (17–20)	13 (12–14)	48 (45–55)
Healthy weight (*n* = 311)	16 (13–18)	4 (3–5)	18 (16–20)	13 (12–14)	50 (46–53)
Overweight (*n* = 93)	14 (13–16)	4 (3–5)	19 (18–20)	13 (12–15)	50 (47–57)
Obesity (*n* = 75)	18 (15–21)	4 (3–5)	20 (19–21)	14 (13–15)	55 (51–58)
*p (KW)*	*< 0.001*	*0.963*	*< 0.001*	*< 0.001*	*< 0.001*
***Workshop attendance (K5)***					
Not attended (*n* = 502)	15 (12–17)	4 (3–4)	18 (16–20)	13 (12–14)	50 (46–55)
Attended (*n* = 75)	21 (19–23)	5 (4–5)	19 (19–20)	14 (13–15)	51.5 (46–57)
*p (MWU)*	*< 0.001*	*< 0.001*	*< 0.001*	*< 0.001*	*0.814*
***Cultural-value alignment with FP (BAC4)***					
Neutral / Not aligned (*n* = 141)	13 (10–16)	4 (3–4)	16 (14–17)	13 (12–13)	49 (46–52)
Fully / Partially aligned (*n* = 436)	16 (13–19)	4 (3–5)	19 (18–20)	13 (12–15)	51 (46–57)
*p (MWU)*	*< 0.001*	*0.002*	*< 0.001*	*< 0.001*	*0.001*

Data are median (Q1-Q3). K, Knowledge & Education (range 8 to 23); AP, Access & Practice (2 to 7); BAC, Beliefs, Attitudes & Cultural Norms (10 to 23); LHS, Lifestyle & Health Status (7 to 18); Overall = pooled 23-item score. Crude p-values from Mann-Whitney U (MWU) for two-group and Kruskal-Wallis (KW) for three or more group comparisons. Crude differences by professional role are descriptive only because students and healthcare workers differ on every demographic indicator (Table 1). Adjusted p-values from multivariable linear regression of each domain on role, with covariates age, education, marital status, residence, and BMI, are reported in the Role row; once these are accounted for, role is not independently associated with any domain (Section “Crude variation in domain scores by role, training, and cultural strata”). FP = family planning; BMI = body mass index

### Factors associated with high counselling confidence

In univariable analysis (Table 4), several demographic, educational, cultural, and contextual factors were significantly associated with high counselling confidence. The strongest unadjusted association was prior workshop attendance (uOR 12.80; 95% CI 6.71 to 24.44), followed by cultural-value alignment with family planning (uOR 6.73; 3.82 to 11.88), comprehensive curriculum coverage (uOR 3.25), city residence (uOR 3.08), guideline familiarity (uOR 3.06), postgraduate education (uOR 2.90), and above-median knowledge (uOR 2.85). Active healthcare workers status was also positively associated in unadjusted analysis (uOR 1.76).

In the multivariable model (*N* = 577; pseudo R² = 0.226; likelihood-ratio *p* < 0.001), high counselling confidence was most strongly associated with workshop attendance (aOR 10.78; 4.92 to 23.62; *p* < 0.001), cultural-value alignment with family planning (aOR 6.10; 3.28 to 11.35; *p* < 0.001), city residence (aOR 3.21; 2.04 to 5.06; *p* < 0.001), and above-median knowledge (aOR 1.71; 1.05 to 2.79; *p* = 0.031). These findings indicate that confidence was more closely associated with modifiable educational and cultural factors, alongside the contextual factor of urban residence, than with professional role alone.

**Fig. 1:**
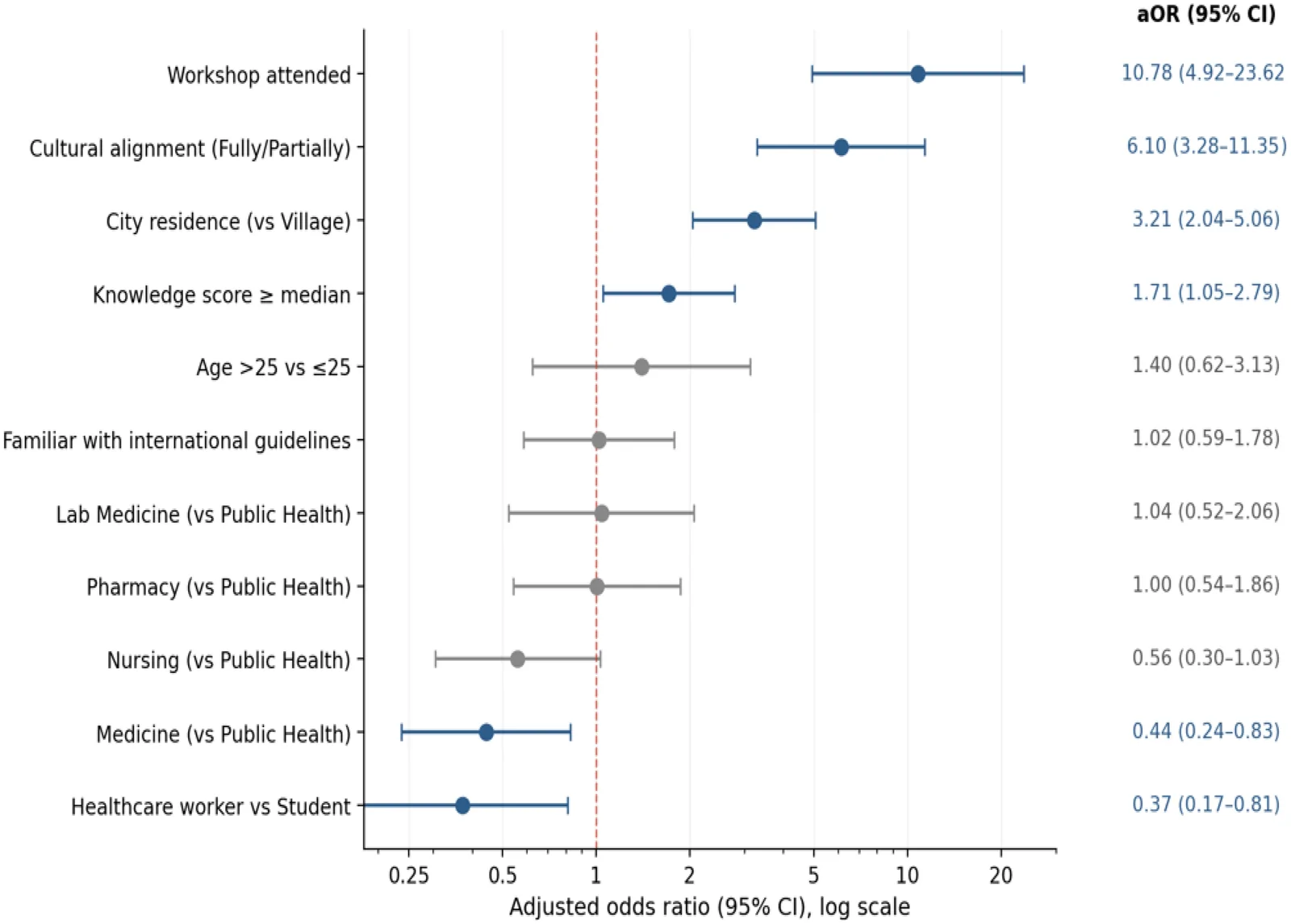
Adjusted odds ratios (aORs) and 95% confidence intervals from the multivariable logistic regression model for high self-rated counselling confidence (*N* = 577). Values above 1 indicate higher odds, and values below 1 indicate lower odds, of reporting being “very confident” in cross-cultural fertility counselling. Statistically significant estimates (*p* < 0.05) are shown in dark blue and non-significant estimates in grey. The dashed vertical line indicates no association (aOR = 1). McFadden’s pseudo R² = 0.226; likelihood-ratio *p* < 0.001

Healthcare workers status showed a negative adjusted association with high confidence in the full model (aOR 0.37; 0.17 to 0.81; *p* = 0.013), and respondents in Medicine had lower adjusted odds than the Public Health reference group (aOR 0.44; 0.24 to 0.83; *p* = 0.010). Variance Inflation Factors for all predictors were below 5 (maximum 2.80), indicating no problematic multicollinearity (Table 4). A hierarchical two-block logistic regression clarified the trajectory of the healthcare workers coefficient: in the univariable model it was positive (uOR 1.76; 1.22 to 2.54; *p* = 0.002); in Block 1 (demographic variables only) it became non-significant (aOR 0.86; 0.46 to 1.61; *p* = 0.639); and in Block 2 (with educational and cultural mediators added) it became significantly negative (aOR 0.37; *p* = 0.013). The Block 2 mediators collectively improved model fit substantially (likelihood-ratio χ²(4) = 124.40, *p* < 0.001; ΔPseudo R² = 0.164). This pattern confirms that the crude positive association between professional role and confidence was explained by demographic differences, and the negative adjusted association reflects model conditioning on variables that may partly mediate the relationship between professional development and counselling confidence. Adjusted estimates from the full model are shown in Fig. 1; the hierarchical model results are presented in Supplementary Table S1.

**Table 4:** Univariable and multivariable logistic regression of factors associated with high self-rated counselling confidence (*N* = 577)

Variable	*n* (%)	uOR	95% CI	*p*	aOR	95% CI	*p*	VIF
Workshop attended (Yes vs. No)	75 (13.0)	12.80	6.71–24.44	< 0.001	10.78	4.92–23.62	< 0.001	1.47
Cultural-value alignment	436 (75.6)	6.73	3.82–11.88	< 0.001	6.10	3.28–11.35	< 0.001	1.10
Curriculum coverage (Comprehensive)	129 (22.4)	3.25	2.17–4.87	< 0.001	-	-	-	-
Residence (City vs. Village)	304 (52.7)	3.08	2.15–4.42	< 0.001	3.21	2.04–5.06	< 0.001	1.23
Guideline familiarity (Full)	126 (21.8)	3.06	2.04–4.59	< 0.001	1.02	0.59–1.78	0.944	1.46
Education (Postgraduate)	66 (11.4)	2.90	1.72–4.89	< 0.001	-	-	-	-
Knowledge ≥ median	333 (57.7)	2.85	1.97–4.12	< 0.001	1.71	1.05–2.79	0.031	1.41
Age (> 25 vs. ≤ 25 years)	129 (22.4)	2.23	1.50–3.33	< 0.001	1.40	0.62–3.13	0.419	2.79
Marital (Married/Other vs. Single)	144 (25.0)	2.72	1.85–4.01	< 0.001	-	-	-	-
Healthcare workers (vs. Student)	171 (29.6)	1.76	1.22–2.54	0.002	0.37	0.17–0.81	0.013	2.80
BMI: Obesity (vs. Healthy weight)	75 (13.0)	3.21	2.02–5.10	< 0.001	-	-	-	-
BMI: Overweight (vs. Healthy weight)	93 (16.1)	1.04	0.64–1.69	0.869	-	-	-	-
BMI: Underweight (vs. Healthy weight)	98 (17.0)	0.48	0.28–0.82	0.008	-	-	-	-
Nursing (vs. Public Health)	117 (20.3)	1.29	0.85–1.96	0.227	0.56	0.30–1.03	0.061	1.54
Pharmacy (vs. Public Health)	102 (17.7)	0.95	0.61–1.49	0.830	1.00	0.54–1.86	0.989	1.40
Laboratory Medicine (vs. Public Health)	78 (13.5)	0.92	0.56–1.52	0.751	1.04	0.52–2.06	0.914	1.47
Medicine (vs. Public Health)	119 (20.6)	0.71	0.46–1.10	0.129	0.44	0.24–0.83	0.010	1.44

Outcome: “very confident” in cross-cultural fertility counselling (yes/no). uOR = unadjusted odds ratio; aOR = adjusted odds ratio; CI = confidence interval; VIF = variance inflation factor. Variables not entered in the multivariable model are shown with a dash (-). The multivariable model included 11 variables: workshop attendance, cultural-value alignment, city residence, knowledge ≥ median, healthcare workers status, age > 25 years, guideline familiarity, and speciality (four dummies with Public Health as reference). Model fit: Pseudo R² = 0.226, likelihood-ratio *p* < 0.001. All VIF < 5 (maximum 2.80), indicating no problematic multicollinearity

### Readiness-confidence profiles

Cross-classification of above- versus below-median Knowledge with high versus lower confidence identified four profiles: calibrated high readiness (*n* = 153; 26.5%), under-confident readiness (*n* = 180; 31.2%), potential overconfidence (*n* = 56; 9.7%), and low readiness (*n* = 188; 32.6%). The two miscalibrated profiles accounted for 236 respondents (40.9%), indicating mismatch between knowledge level and self-rated confidence in approximately two-fifths of the sample.

Profile distribution differed by role, workshop attendance, cultural-value alignment, residence, and speciality (all χ² p < 0.001). Workshop attendance showed the clearest separation: 84.0% of attendees were in the calibrated profile compared with 17.9% of non-attendees, and no attendee was classified as potentially overconfident. Calibrated readiness was also more frequent among respondents with cultural-value alignment (33.0% vs 6.4%) and city residents (36.5% vs 15.4%). Healthcare workers were more often calibrated than students (45.6% vs 18.5%), while potential overconfidence appeared only among students (13.8%). Public Health respondents were over-represented in the potentially overconfident profile (21.7%), whereas Medicine had the highest under-confident proportion (37.8%). These profiles are exploratory and descriptive. The median-Knowledge threshold used to distinguish ‘higher’ from ‘lower’ knowledge is an analytical convenience and should not be interpreted as a validated clinical competence cut-off. Whether potential overconfidence, defined here as high self-rated counselling confidence despite lower knowledge-domain scores, translates into clinically unsafe counselling behaviour cannot be determined from this cross-sectional design; future studies pairing survey-based profiles with Objective Structured Clinical Examination (OSCE) or standardised-patient assessment data would be needed to establish clinical relevance.

### Perceived determinants, barriers, and curriculum endorsement

Respondents most often identified lifestyle and dietary habits as the main local fertility determinant (52.9%), followed by cultural beliefs (25.5%), economic factors (14.4%), and environmental factors (6.8%). The leading perceived barriers to reproductive-health service access were lack of services in rural areas (42.8%) and lack of awareness (31.7%), whereas cultural stigma (14.0%) and cost (10.4%) were reported less frequently. Support for curricular integration was high: 68.5% endorsed fertility-related curriculum integration to a great extent, 27.4% to some extent, and 4.2% did not endorse it. Self-rated counselling confidence varied modestly by discipline, ranging from 30.3% among Medicine respondents to 41.0% among Nursing respondents. These descriptive findings provide context for the training, residence, and knowledge gradients reported in the regression analysis.

## Discussion

In this cross-sectional study of 577 healthcare students and active professionals across five disciplines in Al-Baha Province, fertility-care readiness was uneven and was associated mainly with training exposure, cultural-value alignment, residence, and knowledge. Only 13.0% had ever attended a fertility/RH workshop, only 21.8% reported full familiarity with international RH guidelines, and only 36.2% rated themselves as very confident in cross-cultural fertility counselling, indicating clear supply-side gaps. To our knowledge, this is the first multidisciplinary supply-side workforce study from a transitional Saudi province; it complements the demand-side evidence from women in the same setting [22], and addresses a gap in Saudi KAP literature that has largely focused on community samples or specialist groups in central regions [9, 12, 14, 21]. 

Training exposure was the largest correlate of confidence (aOR 10.78) and is also the most actionable. Structured workshops can be implemented faster and more flexibly than full curricular reform, and could plausibly cover guideline review, common counselling scenarios, referral pathways for assisted reproductive technologies, and culturally sensitive communication strategies. Although the cross-sectional design prevents causal interpretation, the strong association between workshop exposure and confidence supports the case for prospective evaluation of structured fertility/RH training as a workforce-development strategy in transitional Saudi provinces [27, 28]. The finding that only 20.6% considered IVF availability sufficient highlights a particular need for structured education on referral pathways for assisted reproductive technologies.

Cultural-value alignment with family planning was independently associated with high confidence (aOR 6.10). Around three-quarters of respondents reported full or partial alignment, but the residual quarter who were neutral or non-aligned had substantially lower odds of confident counselling. Cultural alignment in this study is not framed as a barrier; rather, it is a marker of the provider’s ability to reconcile professional guidance with local cultural and family-centred expectations. Religious and cultural perspectives in Saudi Arabia are largely permissive of family planning while emphasising marital, familial, and ethical considerations [15, 16, 18–20], so the observed gap is more plausibly attitudinal than doctrinal. Curricular and continuing-professional-development elements that surface and address misalignment, including reflective practice, ethics seminars, and culturally grounded clinical scenarios, would respond directly to this finding. This finding aligns with evidence from other MENA-region provider studies showing that provider–client dynamics and sociocultural context substantially shape the delivery of reproductive-health counselling [29]. 

City residence was independently associated with higher counselling confidence (aOR 3.21) and with higher Knowledge scores. This urban-rural gradient may reflect more frequent training opportunities, greater clinical exposure, easier access to referral pathways and guidelines, and richer informal information networks in the provincial capital [30]. The pattern is consistent with the demand-side evidence from Albanghali et al., which highlighted differential RH-information access across residence categories in the same province [22]. Targeted capacity building beyond urban centres, including mobile training, tele-mentoring, and structured rural placements, could begin to close the gap and align with the geographic equity priorities of Vision 2030.

The role finding requires careful interpretation. In crude analyses, healthcare workers showed higher readiness scores and higher raw confidence than students (45.6% vs. 32.3% very confident). After adjustment for age, residence, and speciality alone (Block 1 of the hierarchical model), healthcare workers status was no longer associated with confidence (aOR 0.86; *p* = 0.639), indicating that the crude role difference was largely explained by demographic, educational, and contextual differences between students and healthcare workers. The coefficient became significantly negative only when educational and cultural mediators were added (Block 2 aOR 0.37; *p* = 0.013), suggesting that the negative adjusted association may be an artefact of conditioning on variables, particularly workshop attendance and cultural-value alignment, that partly mediate the pathway from professional experience to counselling confidence. This interpretation is supported by the substantially improved model fit when mediators were added (likelihood-ratio χ² = 124.40; *p* < 0.001). Because the negative adjusted estimate most plausibly reflects conditioning on mediating variables rather than a genuine inverse independent effect of professional experience on counselling confidence, the HCW coefficient in Block 2 should not be used as a basis for policy-level inference about the relative readiness of healthcare workers versus students. Professional role alone is therefore an insufficient proxy for fertility-care readiness; what matters is what providers have been trained to do and how they reconcile professional and cultural expectations. Self-rated confidence is an imperfect proxy for objective counselling competence: systematic reviews of physician self-assessment show only modest correlations with externally measured performance, with a subset of studies documenting overconfidence among lower-performing practitioners [31]. This underscores the importance of pairing self-report data with objective assessment in future workforce studies.

Medical respondents had lower adjusted odds of high confidence than the Public Health reference group (aOR 0.44). This may reflect more cautious self-assessment among medical respondents because of greater awareness of clinical complexity. Conversely, Public Health respondents may feel more comfortable with counselling and community communication despite lower fertility-specific knowledge scores. Low preconception-health knowledge among Gulf-region medical students has also been documented, with a UAE study reporting a mean knowledge score of 7.5 out of 24 among early-year medical students, indicating that fertility-related knowledge gaps in this workforce are not unique to Al-Baha [32]. The readiness-confidence profile analysis (Section “Readiness-confidence profiles”) supports this reading: potential overconfidence was confined to students and was disproportionately concentrated among Public Health respondents (21.7%), the group with the lowest fertility-specific knowledge scores but the strongest orientation toward community communication. These disciplinary differences are best read as descriptive starting points for tailored curricular design rather than as judgements of competence.

Several implications follow for Saudi workforce development under Vision 2030 and the Health Sector Transformation Program. First, structured fertility and RH workshops should be embedded into pre-service curricula and continuing professional development across the five disciplines studied, building on near-universal stakeholder endorsement (95.8%). Second, culturally responsive counselling modules and guideline-based training should be available to both students and active professionals, with explicit attention to reconciling professional guidance with local cultural expectations. Third, structured referral-pathway education would address the access concerns raised in Section “Item-level readiness across the four domains”, particularly for assisted reproductive technologies. Fourth, capacity building should extend beyond provincial capitals through targeted rural outreach. Fifth, future studies should pair self-reported confidence with objective assessments of counselling competence and evaluate structured training interventions longitudinally.

Strengths include a multidisciplinary sample drawn from the entire current and emerging healthcare workforce of one transitional Saudi province, an analysis plan that explicitly distinguishes descriptive from adjusted findings, and an analytic strategy that pairs supply-side evidence with the demand-side evidence on women in the same province. Limitations are important. The cross-sectional design precludes causal inference, including for workshop attendance. Convenience sampling and university-affiliated recruitment may limit generalisability. The use of convenience sampling through institutional distribution lists may have introduced selection bias toward more digitally engaged or motivated respondents, and the absence of a defined sampling frame means that a formal response rate cannot be calculated. The principal outcome was self-rated confidence, which cannot be equated with objective clinical competence. This is a fundamental limitation of survey-based readiness assessment. Future provincial workforce studies should pair self-report instruments with objective measures of counselling competence, such as Objective Structured Clinical Examinations (OSCEs) or standardised-patient simulations, to establish whether self-rated confidence maps onto operationally measurable counselling skill. Similarly, the readiness-confidence profiles derived in Section “Readiness-confidence profiles” rely on an arbitrary median-split of the Knowledge score and should be regarded as exploratory descriptors rather than validated clinical risk categories. Internal consistency was modest for the AP, BAC, and LHS subscales, so item-level findings are reported alongside domain scores. Residual confounding cannot be ruled out, and the hierarchical logistic regression confirmed that the negative adjusted coefficient for healthcare workers status emerged only when educational and cultural mediators were added to the model (Block 1 aOR 0.86, non-significant; Block 2 aOR 0.37, *p* = 0.013), consistent with over-adjustment by conditioning on mediators. Variance Inflation Factors were all below 5 (maximum 2.80), ruling out multicollinearity as an explanation for the coefficient inversion. The sample was heterogeneous, with substantial demographic differences between students and healthcare workers. These differences limited the interpretation of crude role comparisons and required adjusted analyses; similarly, while professional experience in years was captured (Table 1), it was not incorporated as a continuous predictor in the regression models, which may have attenuated or obscured experience-related gradients in counselling confidence. The single-province setting means replication across other transitional Saudi provinces is needed.

## Conclusion

Fertility-care readiness among healthcare students and active professionals in Al-Baha was uneven and was shaped more by modifiable educational and cultural factors, principally workshop attendance, cultural-value alignment, and knowledge level, as well as the contextual gradient of urban versus rural residence, than by role status alone. Workshop attendance, cultural-value alignment with family planning, city residence, and higher knowledge were the clearest correlates of high self-rated counselling confidence. Although healthcare workers showed higher crude readiness scores than students, this advantage was largely explained by demographic and exposure differences. Self-rated confidence is not a direct proxy for objective clinical competence. These findings support structured fertility and reproductive-health training, culturally responsive counselling education, and targeted capacity building beyond urban centres as supply-side enablers of Vision 2030 RH objectives.

## Supplementary Information

Below is the link to the electronic supplementary material.

Supplementary Material 1

## Data Availability

The dataset generated and/or analysed during the current study are available from the corresponding author on reasonable request.

## Abbreviations

AP: Access and Practice
aOR: Adjusted Odds Ratio
BAC: Beliefs, Attitudes and Cultural Norms
K: Knowledge and Education
KAP: Knowledge, Attitudes and Practices
LHS: Lifestyle and Health Status
RH: Reproductive Health
