# Supplementary material for: Fertility-care readiness among healthcare students and workers in Al-Baha, Saudi Arabia: a cross-sectional study
Source: Contracept Reprod Med. 2026 Jul 22;11:49. doi: 10.1186/s40834-026-00467-7 (PMC13679151; doi:10.1186/s40834-026-00467-7)
Supplement: Supplementary file 1 [file 40834_2026_467_MOESM1_ESM.docx]

**Supplementary Table S1. Hierarchical two-block logistic regression model for high self-rated counselling confidence (N = 577).**

***Block 1: Demographic and structural variables***

| **Variable** | **Block 1 aOR** | **95% CI** | **p** | **Block 2 aOR** | **95% CI** | **p** |
| --- | --- | --- | --- | --- | --- | --- |
| Healthcare worker (vs Student) | 0.86 | 0.46–1.61 | 0.639 | 0.37 | 0.17–0.81 | 0.013 |
| Age >25 years (vs ≤25) | 1.86 | 0.94–3.67 | 0.073 | 1.40 | 0.62–3.13 | 0.419 |
| City residence (vs Village) | 2.77 | 1.87–4.10 | <0.001 | 3.21 | 2.04–5.06 | <0.001 |
| Medicine (vs Public Health) | 0.73 | 0.43–1.23 | 0.232 | 0.44 | 0.24–0.83 | 0.010 |
| Nursing (vs Public Health) | 0.87 | 0.52–1.46 | 0.604 | 0.56 | 0.30–1.03 | 0.061 |
| Pharmacy (vs Public Health) | 1.13 | 0.66–1.94 | 0.657 | 1.00 | 0.54–1.86 | 0.989 |
| Laboratory Medicine (vs Public Health) | 0.83 | 0.44–1.54 | 0.549 | 1.04 | 0.52–2.06 | 0.914 |

***Block 2: Educational and cultural mediators (added)***

| **Variable** | **Block 1 aOR** | **95% CI** | **p** | **Block 2 aOR** | **95% CI** | **p** |
| --- | --- | --- | --- | --- | --- | --- |
| Workshop attended (Yes vs No) | - | - | - | 10.78 | 4.92–23.62 | <0.001 |
| Cultural-value alignment | - | - | - | 6.10 | 3.28–11.35 | <0.001 |
| Knowledge ≥ median | - | - | - | 1.71 | 1.05–2.79 | 0.031 |
| Full guideline familiarity | - | - | - | 1.02 | 0.59–1.78 | 0.944 |

**Block 1:** Pseudo R² = 0.062; AIC = 725.0. **Block 2:** Pseudo R² = 0.226; AIC = 608.6. LR test Block 2 vs Block 1: χ²(4) = 124.40, p < 0.001. ΔPseudo R² = 0.164.

**Key finding:** HCW uOR = 1.76 (univariable, positive) → aOR = 0.86 (Block 1, non-significant, p = 0.639) → aOR = 0.37 (Block 2, significant negative, p = 0.013). The crude positive association disappears with demographic adjustment; the inversion occurs only when mediators are added.
